# Conformational Transition Pathway in the Activation Process of Allosteric Glucokinase

**DOI:** 10.1371/journal.pone.0055857

**Published:** 2013-02-07

**Authors:** Min Huang, Shaoyong Lu, Ting Shi, Yaxue Zhao, Yingyi Chen, Xiaobai Li, Xinyi Liu, Zhimin Huang, Jian Zhang

**Affiliations:** 1 Department of Pathophysiology, Key Laboratory of Cell Differentiation and Apoptosis of Chinese Ministry of Education, Shanghai JiaoTong University, School of Medicine, Shanghai, China; 2 Shanghai Key Laboratory for Tumor Microenvironment and Inflammation, Shanghai JiaoTong University, School of Medicine, Shanghai, China; Uni. of South Florida, United States of America

## Abstract

Glucokinase (GK) is a glycolytic enzyme that plays an important role in regulating blood glucose level, thus acting as a potentially attractive target for drug discovery in the treatment of diabetes of the young type 2 and persistent hyperinsulinemic hypoglycemia of infancy. To characterize the activation mechanism of GK from the super-open state (inactive state) to the closed state (active state), a series of conventional molecular dynamics (MD) and targeted MD (TMD) simulations were performed on this enzyme. Conventional MD simulation showed a specific conformational ensemble of GK when the enzyme is inactive. Seven TMD simulations depicted a reliably conformational transition pathway of GK from the inactive state to the active state, and the components important to the conformational change of GK were identified by analyzing the detailed structures of the TMD trajectories. In combination with the inactivation process, our findings showed that the whole conformational pathway for the activation-inactivation-activation of GK is a one-direction circulation, and the active state is less stable than the inactive state in the circulation. Additionally, glucose was demonstrated to gradually modulate its binding pose with the help of residues in the large domain and connecting region of GK during the activation process. Furthermore, the obtained energy barriers were used to explain the preexisting equilibrium and the slow binding kinetic process of the substrate by GK. The simulated results are in accordance with the recent findings from the mutagenesis experiments and kinetic analyses. Our observations reveal a complicated conformational process in the allosteric protein, resulting in new knowledge about the delicate mechanisms for allosteric biological macromolecules that will be useful in drug design for targeting allosteric proteins.

## Introduction

Glucokinase (GK) is a glycolytic enzyme that plays an important role in blood sugar regulation related to glucose utilization and metabolism in the liver and pancreatic cells (1). GK controls the plasma glucose levels (2,3), and abnormal GK has been associated with the pathogenesis of diabetes of the young type 2 (MODY2) and persistent hyperinsulinemic hypoglycemia of infancy (PHHI) (4–7). The crystal structures of GK in the closed state (active state) and super-open state (inactive state) have been solved by X-ray crystallography, indicating that GK exhibits a global conformational transition between the active and inactive states. Such a global alteration in enzyme conformation may be associated with the special allosteric characteristics of GK (8). Thus, a rigorous mechanistic study of the global conformational transition is critical to understanding the regulation mechanism of GK and to develop new therapeutic approaches for metabolic diseases such as MODY2 and PHHI.

Recently, by using a molecular dynamics (MD) simulation method, we obtained an inactivation pathway for the large conformational transition of GK from the closed state to the super-open state when the glucose concentration is insufficient (9). The overall conformational transition includes three stages, and the three likely stable intermediate states were identified by free energy scanning for snapshots throughout the pathway. The computational predictions were verified by mutagenesis and enzymatic kinetic analysis (9–11). These studies facilitate our understanding of the allosteric mechanism of GK, particularly explaining the sigmoidal kinetic effect of GK (12). However, a reverse large-scale conformational movement of GK activation, propagating from the inactive state towards the active state, is induced for binding and catalyzing substrates when the glucose concentration is increased (10). This is the process that is necessary for GK to perform its function as glucose sensor. Thus, elucidating the key features of the conformational changes of GK that are relevant to its activation will provide insights into the entire allosteric mechanism of GK. Computational simulation, with its details of atomic movements, can be used for investigating such features and the mechanism of GK activation.

Here, we report the study of the conformational transition of GK involved in its activation by using a series of conventional molecular dynamics (MD) and targeted MD (TMD) simulations. By running the simulations on GK, we found a specific conformational ensemble of the inactive state and a consistent conformational transition pathway from the inactive state to the active state. The overall conformational transition includes three stages, and the components that are relevant to the conformational change of GK were addressed by analyzing the snapshots from the TMD trajectories. The simulated results are in accordance with recent findings from mutagenesis experiments and related kinetic studies. In combination with the inactivation process (9), we conclude that the conformational pathway of GK for the activation-inactivation-activation cycle is a one-direction circulation. These observations are important for understanding the entire mechanism of GK regulation and for designing compounds that target GK function for the treatment of metabolic diseases.

## Materials and Methods

### Simulation Systems

Initial coordinates for the closed and super-open states of GK were taken from X-ray crystal structures (Protein Data Bank ID codes 1V4S and 1V4T) (8). The missing residues were repaired by using the loop search method in the Homology module of Insight II (Accelrys, San Diego, CA). For the simulations of GK in aqueous solution, the protein was first put into a suitable sized box, in which the minimal distance from the protein to the box wall was 1.5 nm. Then, the box was solvated with the TIP3P water molecules (13), and the protein/water system was subjected to energy minimization. Afterwards, counterions were added to the system to provide a neutral simulation system, and the whole system was subsequently minimized again.

### MD Simulations

MD simulations were carried out by using the AMBER package (Version 8.0) with constant temperature, constant pressure (NPT), and periodic boundary conditions. The Amber Parm99 force field (14) was applied to the protein. The particle mesh Ewald method (15) was used to calculate the long-range electrostatic interactions. The non-bonded cutoff was set to 12.0 Å, and the non-bonded pairs were updated every 25 steps. The SHAKE method (16) was applied to constrain all covalent bonds involving hydrogen atoms. Each simulation was coupled to a 300 K thermal bath at 1.0 atm of pressure (1 atm = 101.3 kPa) by applying the algorithm developed by Berendsen *et al.* (17). The temperature and pressure coupling parameters were both set as 1 ps. An integration step of 2 fs was set up for the MD simulations. Water molecules and protein were coupled separately to a temperature bath at 300 K using a coupling time of 0.1 ps. Finally, a 50-ns conventional MD simulation was performed on the super-open state of GK.

The TMD approach (18,19), implemented in AMBER 8.0, was used to apply a restraint force onto each initial structure to bias the trajectories toward each respective target. A restraint defined in terms of a mass-weighted root mean square deviation (wRMSD) to the final reference structure (target) was applied in the force field as an extra energy term, which was calculated by the following equation:
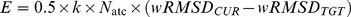
(1)where *k* is the force constant, *N*_atc_ is the number of atoms, *wRMSD_CUR_* is current wRMSD by superposition to the final reference structure, and *wRMSD_TGT_* is the target wRMSD. Four TMD simulations (B1–B4) were performed using a force constant of 0.5 kcal·mol**^−^** ^1^·Å**^−^** ^2^ with different initial velocities, and three simulations (C1–C3) were carried out with larger force constants of 1.0, 1.5, and 2.0 kcal·mol**^−^** ^1^·Å**^−^** ^2^, respectively. A force constant of 0.5 kcal·mol**^−^** ^1^·Å**^−^** ^2^ over 1 ns was found to be sufficient to determine a low-energy path leading from the initial structure (super-open state of GK) to the target structure (closed state of GK).

The TMD simulations were performed by using the Sander module encoded in AMBER (Version 8.0). The integration step for TMD simulation was set to 1 fs. The detailed procedure of TMD simulation has been described by Schlitter *et al.* (20) and used in several recent applications (9,21,22).

### Docking of Glucose to GK

To investigate the binding properties of glucose to GK during its activation process, glucose was docked into a series of GK conformations, which were extracted every 100 ps from the B1 trajectory. The docking was performed using GOLD (23) and targeting the entire protein structure of each GK conformation. For each genetic algorithm (GA) run, a maximum number of 200,000 operations were performed on a population of 5 islands for 100 individuals. Operator weights for crossover, mutation, and migration were set to 95, 95, and 10, respectively. GoldScore, implemented in GOLD, was used as the primary function to evaluate the docked conformations. The highest ranked conformation in the largest cluster from the docking simulations was selected to subject to binding analysis and free energy estimation using X-Score (24). The wRMSD of heavy atoms between the crystal pose (PDB entry: 1V4S) and docked pose of glucose was calculated by the scripts encoded in the DOCK4 program (25).

## Results

**Figure 1:**
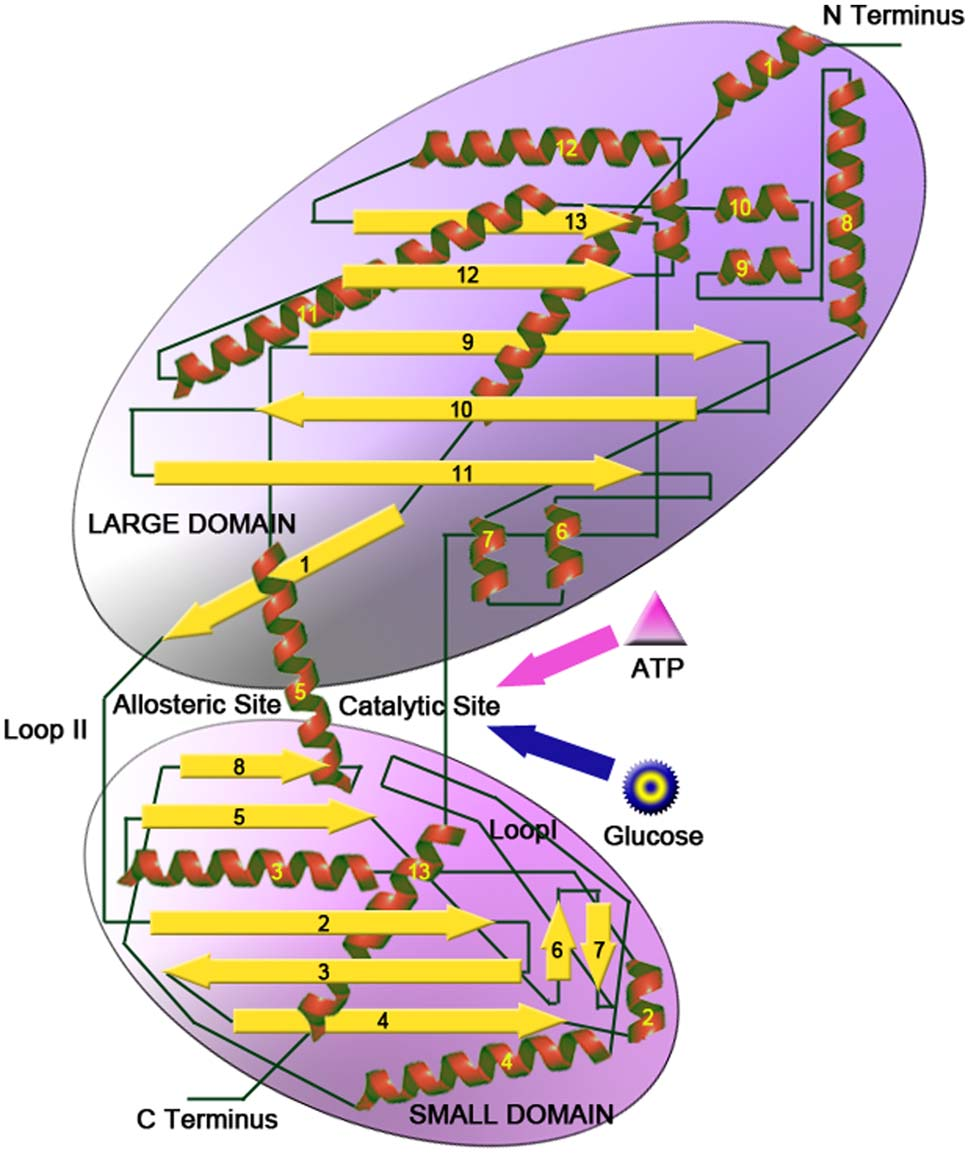
2D topological structure plot of GK.

The main goal of this study was to investigate the activation process of human GK, whose 2D topological structure plot is shown in Figure 1. The activation process of GK is associated with a large-scale conformational change from the super-open state to the closed state. To study this process, three models for conventional MD and TMD simulations were designed. In model **I**, a 50-ns simulation (A1) was conducted to probe the conformational space of GK on the inactive state. In model **II**, four TMD simulations (B1–B4) were performed by using a force constant of 0.5 kcal·mol**^−^** ^1^·Å**^−^** ^2^ with different initial velocities to explore the minimal energy pathway between the super-open state and the closed state. In model **III**, to verify the reliability of the pathway for the conformational alternation of GK, three additional TMD simulations (C1–C3) were carried out with force constants of 1.0, 1.5, and 2.0 kcal·mol**^−^** ^1^·Å**^−^** ^2^, respectively. In total, eight MD simulations were performed on GK. More detailed information for these MD simulations is provided in Tables S1 and S2 in the *Supporting Information*.

### Conformational Ensemble of GK from the Inactive State

Structurally, free GK exists in a super-open form characterized by two separate domains connected by a hinge region. Therefore, the active site of phosphorylation between the large and small domains is not formed, leading to the inactive function of catalysis (8). To explore the conformational change of GK in the activation process, conventional MD simulation from the super-open state was performed as model **I**. The root mean square deviation (RMSD) value along the simulation is shown in the *Supporting Information* Figure S1A, and the time evolution of the cleft angle between the two domains (9) was monitored and is shown in the *Supporting Information* Figure S1B. According to these results, the conformational change of GK was rather slow, and the cleft angle profile underwent a periodic fluctuation from ∼70° to ∼45° during the 50-ns simulation, revealing a conformational ensemble of GK from the inactive state.

### Influence of Different Initial Velocities and Forces to the Activation Pathway of GK

**Figure 2:**
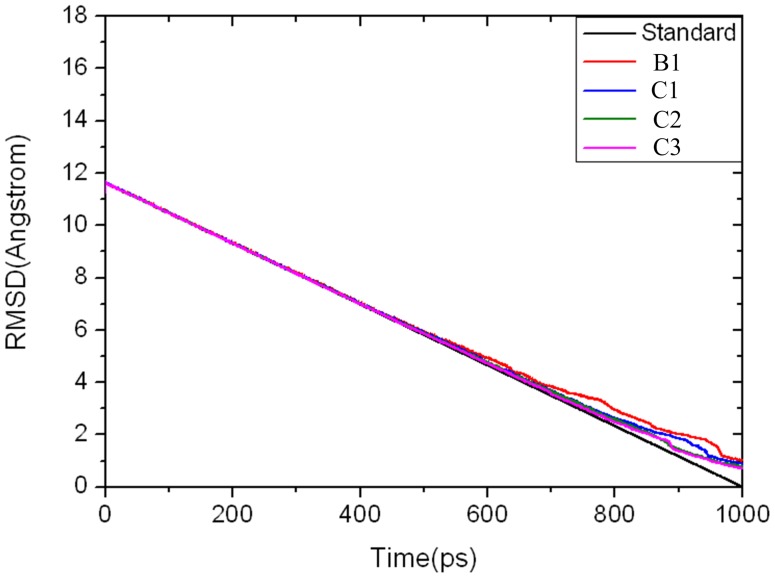
Time evolutions along with the wRMSD of the backbone atoms of GK in the simulated structures compared with the reference structure. Progression of the target wRMSD value is in black; actual values for GK corresponding to the trajectories of B1 and C1-C3 are in red, blue, green, and pink, respectively.

The activation process of GK involves a global alternation from the super-open state to the closed state, which includes a large-scale conformational change that cannot be attainable by conventional MD simulation. Accordingly, TMD, which can accelerate the process of large-scale conformational motion between two existing states, was used in this study to address the potential allosteric pathway of GK. To obtain a reliable pathway for the global conformational transition from the super-open state to the closed state, four TMD simulations (B1–B4) were performed on model **II** under a force constant of 0.5 kcal·mol**^−^** ^1^·Å**^−^** ^2^ with different initial velocities. The four TMD simulations produced almost the same conformational transition pathway, as demonstrated by the wRMSDs among the four trajectories (Table S1 in the *Supporting Information*), indicating that the initial velocities for TMD simulations do not affect the transition pathway. To probe the influence of the force constant on the conformational transition pathway, three additional TMD simulations (C1–C3) were conducted on model **III** under force constants of 1.0, 1.5, and 2.0 kcal·mol**^−^** ^1^·Å**^−^** ^2^, respectively. In comparison with trajectories B1–B4, trajectories C1–C3 did not give qualitatively different results (Table S2 in the *Supporting Information* and Figure 2). All these results suggested that the conformational transition pathway is reliable. In the following discussion, we used the trajectory B1 for analysis.

### Potential Pathway for Global Conformational Transition

**Figure 3:**
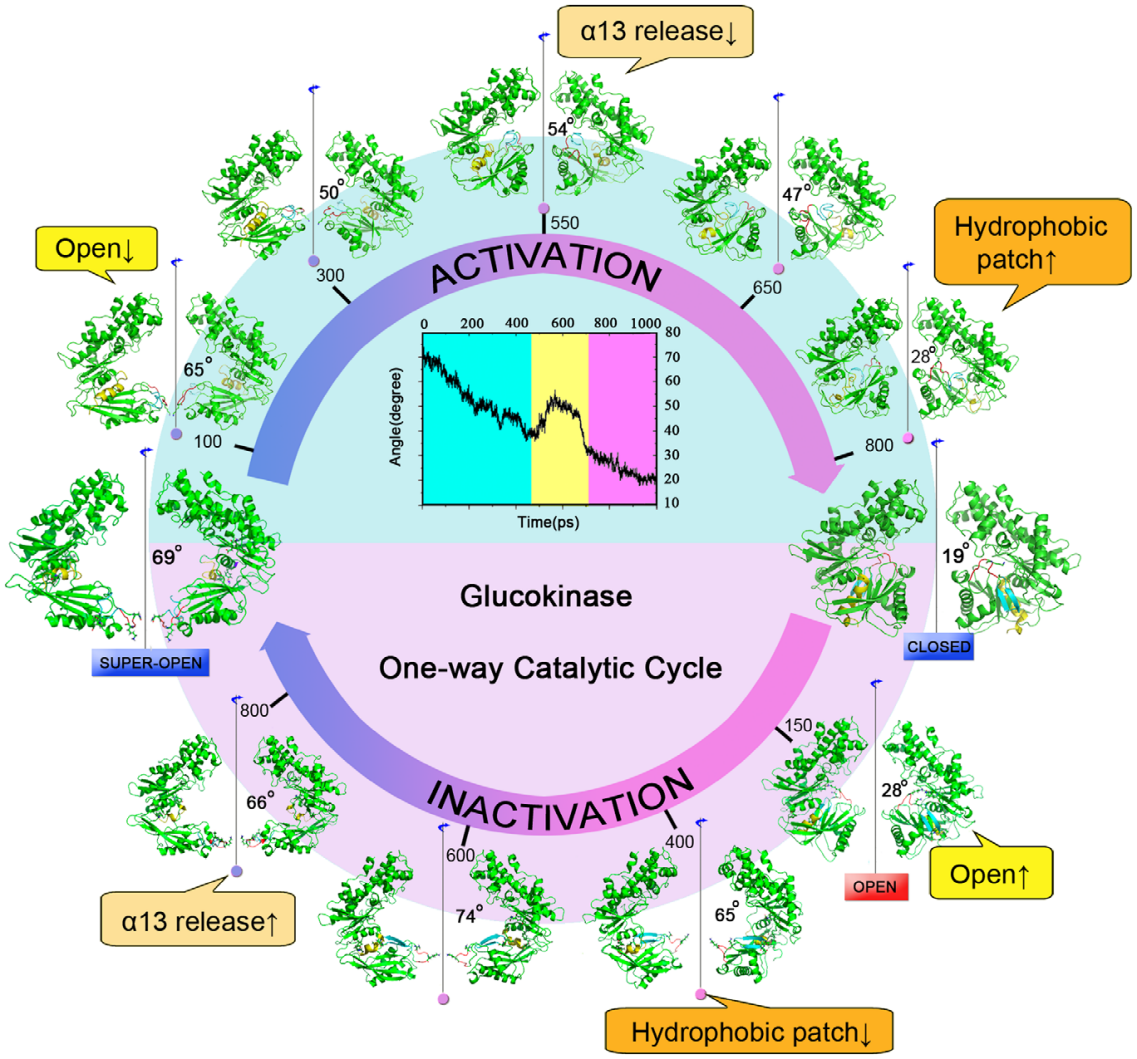
The conformational transition pathway of GK between the super-open state and the closed state. The center image is the time dependence of the cleft angle between the two domains in the process of GK activation. Around the upper semicircle of the center image, five snapshots are extracted from trajectory B1 at the activated times of 100 ps, 300 ps, 550 ps, 650 ps and 800 ps. Two crystal structures of GK in the super-open and closed states are shown at either end of the catalytic cycle, and four snapshots at the inactivated times of 150 ps, 400 ps, 600 ps and 800 ps that were found previously are also shown in the lower semicircle (9). Three important components of conformational change in GK are labeled in comment frames. Loop I between the α4 and β7 segments is in red; the β6 and β7 strands are in cyan; helix α13 is in yellow.

The TMD-simulated conformational transition pathway of GK activation from the super-open state to the closed state is shown in Figure 3. Although the transition occurs continuously, the overall conformational transition can be roughly divided into three stages. The conformational transition began with a stage in which the cleft angle gradually closed down ∼36° during the time period from 0 to 450 ps (denoted ‘open↓’). In this first stage, there was no direct interaction between the large and small domains of GK at the beginning of activation process, and the process was able to go through the first conformational stage smoothly. At the second stage (450–700 ps), the secondary structure of the α13 helix distorted and squashed into incapacious space on the back of the cleft between the large and small domains (denoted ‘α13 release↓’), and the β6 and β7 residues in the small domain continually adjusted their positions within the range of the local space of the cleft after GK underwent a rapid closing process. At ∼660 ps, loop I moved away from the solvent to reorient and started to insert into the cleft. During this stage, the residues in β6 and β7 began to fold their secondary structures into strands. In the third period (700–1,000 ps), the cleft angle reached the closed state, and the β6 and β7 residues completely converted into β strands and were embedded in a hydrophobic pocket found previously (denoted ‘hydrophobic patch↑’) (9). Meanwhile, the tail of α13 helix completely spanned loop II, restored the secondary structure and constructed the allosteric site within α5, β1, β8 and loop II.

### Possible States in the Transition Pathway

**Figure 4:**
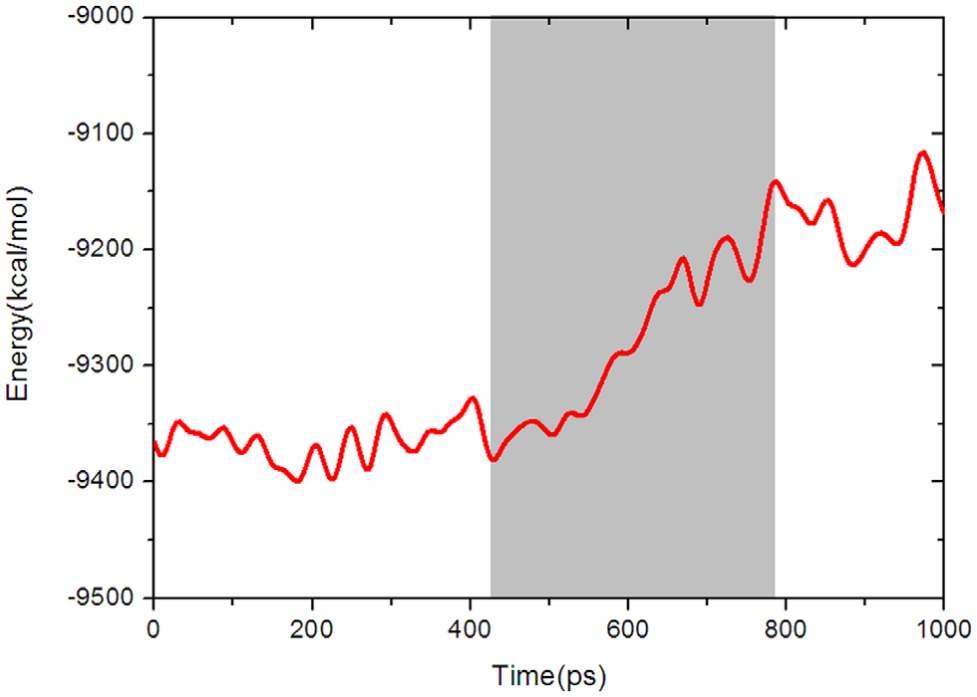
Free energy landscape along the conformational transition pathway of GK activation.

The x-ray crystal structures of GK indicate that the conformational transition from the super-open state to the closed state is a complicated process. According to the mechanism of GK regulation proposed by Kamata *et al.* (8) and the behaviors of other hexokinases, the activation process of GK should undergo a conformational transition from the low-energy inactive state to the high-energy active state through transition state(s) (26). Nevertheless, such types of transition states of GK have not been detected experimentally (8). To explore the possible transition states for GK, the free energy landscape along the conformational transition pathway was calculated by using the MM-PBSA-NMODE method encoded in AMBER (27–31). As shown in Figure 4, there were three energy phases (denoted as P1, P2, and P3) in the pathway at time periods of 0–430, 430–780, and 780–1000 ps, which correspond to the above three conformational transition stages. Conformations in P1 evolved within the low free energy range in the first closing process, and then, the energy gradually ascended as time increased in P2. Finally, high level energy fluctuations were maintained until GK reached its fully closed state. The phase of low energy may facilitate the conformational change of GK from the super-open state, but the following energy barrier greatly delays the process. This is in good agreement with the result of model **I**, in which the limited conformational ensemble (cleft angle from ∼70° to ∼45°) was found using the conventional MD simulation. The energy profile is also consistent with the kinetic analysis that determined GK successively undergoes a rapid and slow phase conformational change on activation (11).

**Figure 5:**
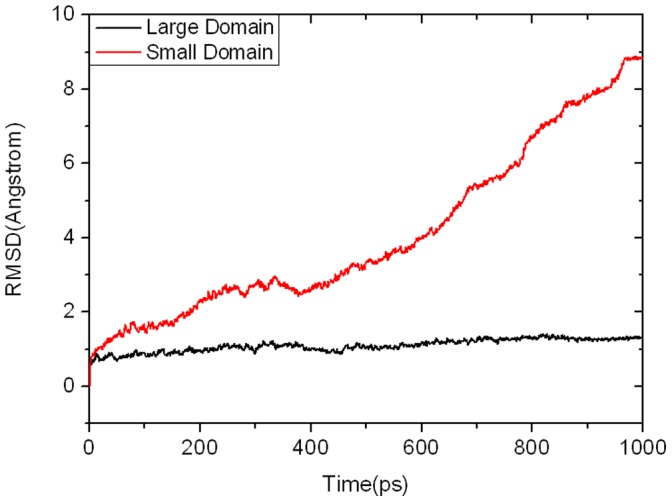
Time dependency of wRMSDs of the large domain (black line) and small domain (red line) in GK during the TMD simulation.

In the activation process of GK, as the cleft angle between the large and small domains decreases (Figure 3), the energy of the system gradually increases (Figure 4). To investigate the contribution of each domain to such changes, the wRMSDs of the large and small domains were respectively monitored along the whole MD process after fitting the snapshots to the corresponding large and small domains of GK. As shown in Figure 5, the wRMSD of the large domain is nearly flat, while that of the small domain is rising. Accordingly, it can be proposed that the large domain of GK moves almost as a rigid body during the conformational transition, whereas the small domain of GK possesses a large and complicated conformational change along the activation process.

### Important Components along the Transition Pathway

**Figure 6:**
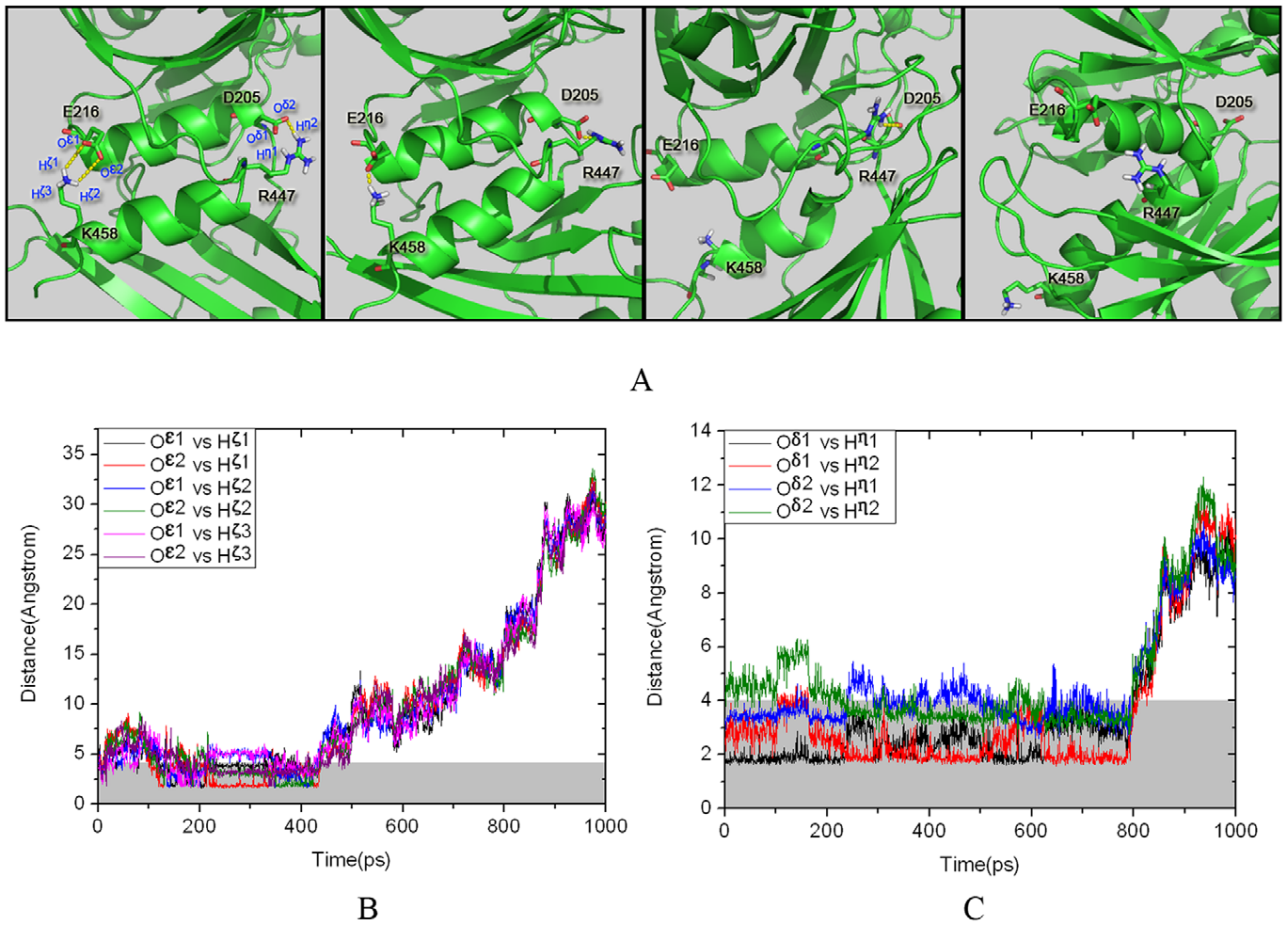
Changes in the hydrogen bonds between residues Asp205 and Arg447 and residues Glu216 and Lys458 during the TMD simulation. (A) Snapshots at 1 ps, 300 ps, 700 ps and 1000 ps. (B) Distances from the O^ε1^ and O^ε2^ atoms in Glu216 to the H^ζ1^, H^ζ2^ and H^ζ3^ atoms in Lys458. (C) Distances from the O^δ1^ and O^δ2^ atoms in Asp205 to the H^η1^ and H^η2^ atoms in Arg447.

At the beginning of the activation process of GK, the cleft angle between the large and small domains shrank (the first stage in Figure 3) with no energy barrier (P1 in Figure 4). This result indicates that the large and small domains smoothly approach each other in the first stage, which was also observed in the 50-ns conventional MD simulation. However, in the second activation stage, the energy of the system increased rapidly as shown in Figure 4. This result reveals that the unfavorable change of interaction may be responsible for the observed energy cost for GK in the transition period. The key to the change seems to derive from the rupture of two pairs of hydrogen bonds between residues Asp205••••••Arg447 and Glu216••••••Lys458 (Figure 6), which stabilize the parallel α13 and α5 in the inactive structure of the super-open state (8). The TMD simulation showed that these hydrogen bonds remain in effect during the first stage of conformational transition (Figure 6B and Figure 6C). After 430 ps, the hydrogen bond between residues Glu216 and Lys458 was ruptured, and the terminus of α13 began to turn away from the parallel α5. At about 780 ps, the hydrogen bond between Asp205 and Arg447 was entirely broken, and the entire α13 helix was embedded into the cavity lined by α5, β3, β4 and β5 from the small domain. Notably, the breaking points of the hydrogen bonds are consistent with the predicted second stage in the pathway of conformational transition, as indicated by the free energy calculation (Figure 4), implying that the energy ascent may be evoked by the rupture of these hydrogen bonds. These results also highlight that the hydrogen bonds play important roles in the conformational transition of GK from the inactive state to the active state.

**Figure 7:**
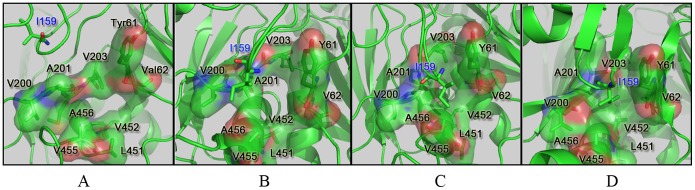
Changes of the hydrophobic pocket and its hydrophobic interaction to Ile159 along the TMD simulation, with snapshots at 900 ps (A), 920 ps (B), 980 ps (C) and 990 ps (D).

The interaction between Ile159 and the “hydrophobic pocket” composed of Tyr61, Val62, Val200, Ala201, Met202, Val203, Leu451, Val452, Val455 and Ala456 serves as a stabilizer to maintain the active state of GK (9). As shown in Figure 7, this process produced three stages in the conformational transition pathway. First, the hydrophobic pocket was incompact, and Ile159 was far away from the pocket until 900 ps (Figure 7A). With the process of GK activation, the hydrophobic residues of the pocket closed up, and the distance between Ile159 to the centroid of the pocket gradually reduced from 920 ps to 980 ps (Figures 7B and 7C). Finally, a compact hydrophobic pocket was formed, which Ile159 completely fitted into after 990 ps (Figure 7D). This hydrophobic interaction has a vital role in GK activation, which has been validated by several mutagenesis studies. For example, the mutants V62M/L, V455M and A456V enhance the hydrophobic interaction with the decrease of the S_0.5_ (concentration of glucose at which glucokinase shows the half activity of *V*_max_) value (8,32,33), while the mutants Y61S, A201R, V203E, V452S and I159A weaken the hydrophobic interaction with the increase of the S_0.5_ values in the kinetic curve of GK (9).

### Binding Regions of Glucose During the Transition Pathway

**Figure 8:**
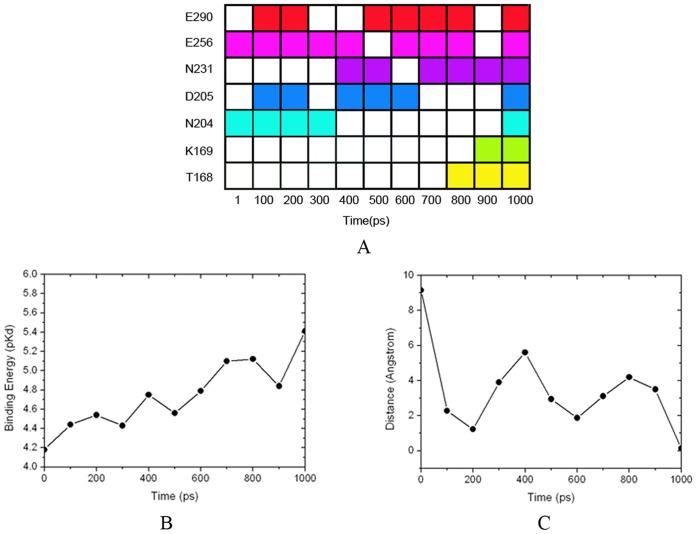
Process of glucose binding to GK. (A) The residues of GK in contact with glucose during the TMD simulation, represented by different colors. The colored frame means glucose has contact with the residue, while the blank frame means glucose does not have contact with the residue. (B) Binding affinity between glucose and GK calculated by X-Score. (C) wRMSD of the heavy atoms between the crystal pose and docked pose of glucose.

The substrate, glucose, first binds to the super-open state of GK and goes along with the large-scale conformational transition of GK activation until the stereochemical environment of the second substrate (ATP) is achieved and catalysis is triggered (8). To investigate the dynamic binding mechanism of glucose to GK during its activation process, a docking simulation was employed to determine the location of glucose in different GK conformations. The binding pocket for glucose catalysis is sandwiched by the large and small domains. At the beginning of the activation process, the pocket was incompletely shaped, with only Asn204 and Glu256 able to fix the glucose through hydrogen bonding interactions with a low binding affinity (Figures 8A and 8B). At that time, the binding pose of glucose was also far away from its catalyzed position, as revealed by the wRMSD (Figure 8C). The docking predictions for the glucose binding positions at GK along with the conformational changes are in agreement with the mutagenesis results. For example, the mutants N204A, E256A and E256K are essentially inactive even at a glucose concentration of 200 mM (32), implying that the binding in the first step is a prerequisite for glucose to be phosphorylated by GK. Then, GK gradually adjusted its binding pocket for glucose, in which the glucose adapted itself to GK after 200 ps in the TMD simulation. Subsequently, the glucose successively formed hydrogen bonds with Asp205, Asn231 and Glu290 in the large rigid domain and connecting region of GK until 800 ps, enhancing its binding affinity in the first and second stages of GK activation (Figure 8A). After that, the small flexible domain moved closer so that it could interact with the glucose, and the binding pocket for glucose was finally formed. Meanwhile, Thr168 and Lys169 of the small domain induced the glucose to obtain its active pose, and the binding affinity of GK for glucose reached its maximum value (p*K*_d_≈5.41), in good agreement with the available kinetic affinity and crystal analysis data (8,32). When GK achieves its closed state with active glucose (Figure 8C), ATP finally binds to GK, triggering the phosphorylation of glucose (8). The energy release from glucose binding and the subsequent ATP binding, to some extent, may compensate for the energy requirement of the conformational transition from the inactive state to the active state of GK.

## Discussion

It is extremely expensive to obtain a full view of large-scale conformational pathway using experimental method or conventional computational simulation. Herein, TMD simulations may provide a general mode to study the process of such conformational transition. The reliability of process depends on the force constant parameter in TMD simulation. Large-scale conformational change in the TMD simulation cannot be driven if the force constant is too small, whereas irrelevant distortion in TMD conformations with high internal energy will be obtained if the force constant is set too large. Therefore, the minimum force constant which can drive TMD simulation is optimal (34). Moreover, in the case of our study, we found that the force constant of 0.2 kcal·mol**^−^** ^1^Å**^−^** ^1^ is appropriate for the inactivation process (9), nevertheless, it is not large enough in the activation process (data not shown) until it increases to 0.5 kcal·mol**^−^** ^1^Å**^−^** ^1^, indicating that the drive of two processes in one cycle could be different and their optimal force constants must be carefully detected.

**Figure 9:**
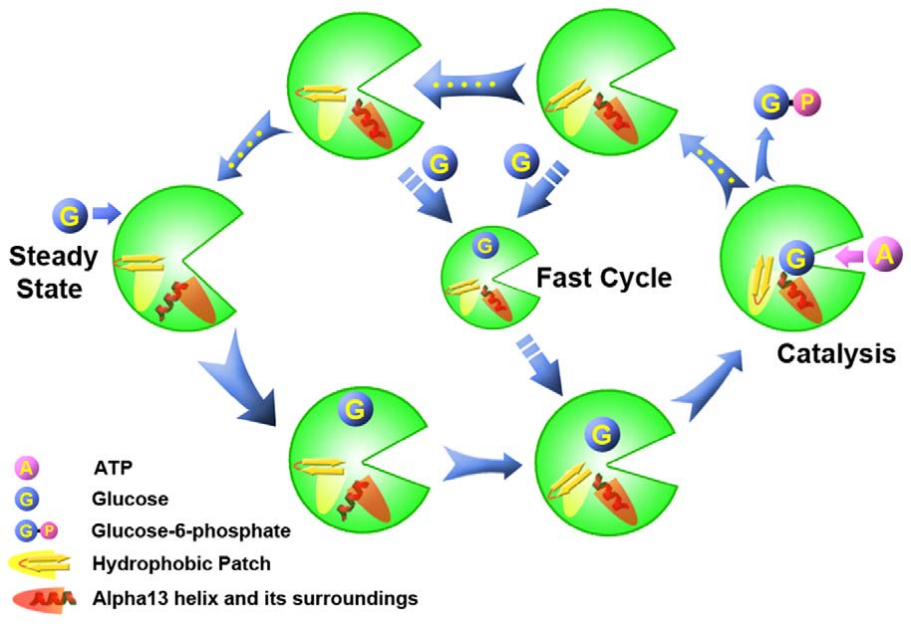
An atomic mechanism for the whole catalytic circulation of GK. The width of the arrow indicates the time scale in the conformational pathway, and yellow dots in the arrow represent the intermediate state of the energy well in the activation-inactivation-activation process.

The inactivation and activation process of GK have been investigated by MD simulations in previous studies as well as the current study (9). Herein, we propose an atomic mechanism for the whole circulation of GK. As shown in Figure 9, during the activation process, the opening cleft between the large and small domains of GK smoothly decreases, followed by the return of the α13 helix from its inactivated state, and finally, GK reaches the closed state with the active conformation of glucose after the “hydrophobic patch” in GK is formed. At this time, ATP enters the cleft of GK and transfers its γ-phosphate to glucose. Once the catalysis by GK is finished, the cleft of GK begins to open (‘open↑’), and the products are released. Three intermediate states delay the opening process of GK, in which the “hydrophobic patch” is broken (‘hydrophobic patch↓’), and the α13 helix is released (‘α13 release↑’). GK returns to the super-open inactivated state if no glucose binds to GK to trigger a “fast cycle” during the intermediate states. The process can be simply described as “steady state-open↓-α13 release↓-hydrophobic patch↑-catalysis-open↑-hydrophobic patch↓-α13 release↑-steady state” (Figure 3), which gives us complete insight into the mechanistic mechanism of GK.

To further validate the reliability of our TMD-generated transition pathways, here the super-open state (PDB ID: 1V4T) and closed state (PDB ID: 1V4S) of GK were additionally submitted to the Molmov morphing server (http://www.molmovdb.org/molmovdb/morph/), which, by performing an adiabatic mapping (a restrained interpolation) between every two conformations, produces a plausible conformational transition pathway between two submitted protein conformations. As shown in Video S1, 8 default intermediate conformers (as output from Molmov) were shown along with the super-open to closed states of GK. Visual inspection of Video S1 definitely corroborated that the secondary structure of the α13 helix was distorted and squashed into incapacious space on the back of the cleft between the large and small domains of GK on the process from the super-open to closed states. In addition, we conducted the nudged elastic band (NEB) (35–37) method (see Text S1) to probe the transition pathway from the super-open state to the closed state of GK. After the optimizations, the 20 images were shown to stretch along with the transition pathway from the super-open to closed states of GK (Video S2). The extracting transition pathway offered a fascinating opportunity to manifest that the opening cleft between the large and small domains of GK smoothly decreased and the dynamics characteristics of the secondary structure of α13 helix resembled the TMD- and Molmov-generated features. The two conformational transition pathways originated from the Molmov morphing server and NEB method therefore match well with the TMD-generated transition pathways, arguing that our TMD-generated conformational transition pathways are reliable.

From the TMD-generated transition pathways, it is clear that the super-open state of GK (*unbound*) is inherently dynamics and can with a certain probability sample the same conformations as observed in the closed state of GK (*bound*), that is, the *bound* protein conformation pre-exists in the ensemble of conformations sampled by the free protein in the absence of ligand. The ligand, glucose, can bind to the *bound* conformation of the unligand GK. This binding interaction does not ‘induce’ a new conformational change; it merely leads to a population shift, in other words, a redistribution of the relative populations of conformational substates that already pre-dispose in solution. Following initial binding through a conformational selection mechanism, it is probable that further conformational changes to the protein structure, especially in the optimization of sidechain and backbone interactions, are induced to orchestrate the protein-ligand interactions. Thus, it is likely that both conformational selection and induced fit play important roles in molecular recognition in GK. The co-existence of both mechanisms is convincingly evident in interactions of many protein-ligand, protein-protein, and protein-DNA interactions.

As an expansion of the mnemonic model (38,39), the “Ligand-Induced Slow Transition (LIST)” model developed by Neet and co-workers (40) provides a more reasonable explanation for the kinetic behavior of GK (41–43), in which a preexisting equilibrium is proposed to involve at least two distinct GK conformers in absence of a substrate, but which is not explicitly understood in dynamic conformations (44–47). Our simulations found that GK conformations in the first transition stage of activation (the time period from 0 to 430 ps, Figure 4) could easily interconvert due to very low energy barriers between them (cleft angle in model **I**), providing structural evidence at an atomic level for the preexisting equilibrium. Meanwhile, steadily increasing the binding affinities of glucose to GK (Figure 8B) during equilibrium also supported the multiple binding conformations of glucose postulated by Antoine (46). Furthermore, the large energy barrier in the second stage (the time period from 430 to 780 ps, Figure 4) retards the conformational interconversion, which is in good agreement with a slow kinetic process from the substrate binding to its catalysis (46). Therefore, a one-direction circulation model of GK could uncover a more detailed molecular mechanism behind the biochemical phenotype.

Allostery is the most direct, rapid and efficient way for the regulation of protein function, ranging from the control of metabolic mechanisms to signal transduction pathways (48). Dysregulations of allosteric systems have been significantly associated with human diseases, such as diabetes, inflammation, and Alzheimer’s disease (49–51). Therefore, understanding the mechanisms of allosteric proteins becomes crucial for diagnosing the increasing number of allosteric diseases and designing novel allosteric modulators for drug discovery (52–54). As a case study, the allosteric mechanism of a one-direction circulation in GK reveals a complicated relationship between structure and function as well as transient targetable conformations for modulators, which provides new knowledge about the complicated mechanic mechanisms of allosteric biological macromolecules.

### Conclusions

GK plays a key role in maintaining glucose homeostasis and might be an important therapeutic target for treating metabolic diseases, such as MODY2 and PHHI. Our study uncovered the activation process of GK through conformational transitions from the super-open state (inactive state) to the closed state (active state). Using TMD simulations, we found that GK undergoes three stages: first, the large and small domains of GK drift toward each other smoothly; second, the hydrogen bonds that restrain α13 in the inactive state are disrupted one after the other, and the helix restores its orientation towards the active state; finally, a crucial hydrophobic interaction between Ile159 and the hydrophobic pocket formed by Tyr61, Val62, Val200, Ala201, Met202, Val203, Leu451, Val452, Val455 and Ala456 helps stabilize the active state of GK. Additionally, the initial binding conformation of glucose was found to adapt to the binding site via its interaction with the residues in the large domain and connecting region of GK during the activation process. The whole conformational pathway in the activation-inactivation-activation mechanism of GK is a one-direction circulation, and the active state is less stable than the inactive state in the one-direction circulation. These results are in agreement with the reported experimental data and might be very useful for deciphering the mechanism governing the conformational transition of GK. Our findings may also provide new knowledge in the study of the complicated mechanic mechanisms of allosteric biological macromolecules and aid in drug design for treating metabolic diseases by targeting allosteric proteins.

## Supporting Information

Figure S1 **Analyses for the conventional MD simulation on GK.** (A) Time dependency of RMSD from free GK in the 50-ns MD simulation. (B) Cleft angle profile along the 50-ns trajectory. The angle is defined by two lines from the Cα atom of Cys233 (hinge residue) to the Cα atoms of Gly229 and Lys169 (9).
(TIF)

Table S1 **wRMSDs between trajectories using different initial velocity under the restraint force of 0.5 kcal·mol^−1^·Å^−2^. wRMSDs calculated on backbone atoms and all atoms are listed on the upper triangle and lower triangle of the table.** Data in the parentheses are variance of wRMSD between two trajectories
(DOC)

Table S2 **wRMSDs between two trajectories using different forces of 0.5, 1.0, 1.5 and 2.0 kcal·mol^−1^·Å^−2^.** wRMSDs calculated on backbone atoms and all atoms are listed on the upper triangle and lower triangle of the table. Data in the parentheses are variance of wRMSD between two trajectories.
(DOC)

Text S1 **The nudged elastic band to locate the transition pathway fro the super-open to closed states.**
(DOC)

Video S1 **The conformational transition pathway of eight GK intermediate conformers between the super-open and closed states obtained by means of Molmov interpolation.** The α13 helix is colored in red.
(MPG)

Video S2 **The conformational transition pathway from the super-open to closed states was generated from the nudged elastic band method.** The small domain, the large domain, and the α13 helix are colored in cyan, orange and red.
(MPG)
