# Supplementary figures and images for: Conformational Transition Pathway in the Activation Process of Allosteric Glucokinase
Source: PLoS One. 2013 Feb 7;8(2):e55857. doi: 10.1371/journal.pone.0055857 (PMC3567010; doi:10.1371/journal.pone.0055857)

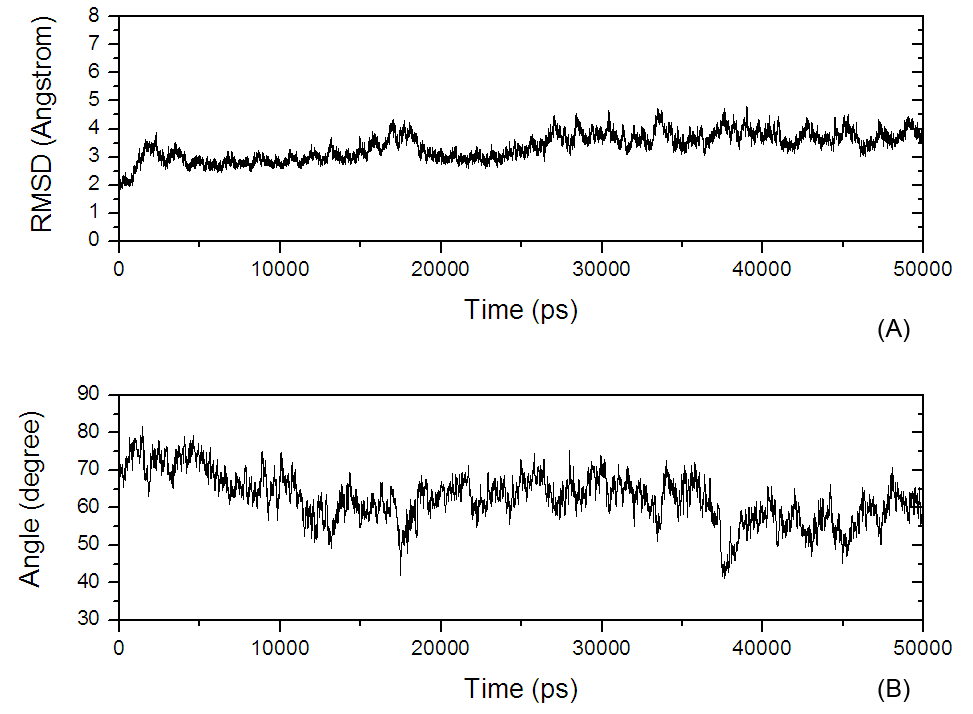

Supplement: Figure S1 — Analyses for the conventional MD simulation on GK. (A) Time dependency of RMSD from free GK in the 50-ns MD simulation. (B) Cleft angle profile along the 50-ns trajectory. The angle is defined by two lines from the Cα atom of Cys233 (hinge residue) to the Cα atoms of Gly229 and Lys169 (9). (TIF) [file pone.0055857.s001.tif]
