# Supplementary material for: Conformational Transition Pathway in the Activation Process of Allosteric Glucokinase
Source: PLoS One. 2013 Feb 7;8(2):e55857. doi: 10.1371/journal.pone.0055857 (PMC3567010; doi:10.1371/journal.pone.0055857)
Supplement: Table S1 — wRMSDs between trajectories using different initial velocity under the restraint force of 0.5 kcal·mol−1·Å−2. wRMSDs calculated on backbone atoms and all atoms are listed on the upper triangle and lower triangle of the table. Data in the parentheses are variance of wRMSD between two trajectories (DOC) [file pone.0055857.s002.doc]

**Table S1*.*** wRMSDs between trajectories using different initial velocity under the restraint force of 0.5 kcal·mol^-1^·Å^-2^. wRMSDs calculated on backbone atoms and all atoms are listed on the upper triangle and lower triangle of the table. Data in the parentheses are variance of wRMSD between two trajectories

|  | B1 | B2 | B3 | B4 |
| --- | --- | --- | --- | --- |
| B1 | 0(0) | 1.55(0.21) | 1.60(0.43) | 1.56(0.45) |
| B2 | 1.56(0.29) | 0(0) | 1.72(0.34) | 1.80(0.29) |
| B3 | 1.61(0.31) | 1.62(0.34) | 0(0) | 1.68(0.33) |
| B4 | 1.64(0.38) | 1.78(0.28) | 1.72(0.36) | 0(0) |
