# Supplementary material for: Conformational Transition Pathway in the Activation Process of Allosteric Glucokinase
Source: PLoS One. 2013 Feb 7;8(2):e55857. doi: 10.1371/journal.pone.0055857 (PMC3567010; doi:10.1371/journal.pone.0055857)
Supplement: Table S2 — wRMSDs between two trajectories using different forces of 0.5, 1.0, 1.5 and 2.0 kcal·mol−1·Å−2. wRMSDs calculated on backbone atoms and all atoms are listed on the upper triangle and lower triangle of the table. Data in the parentheses are variance of wRMSD between two trajectories. (DOC) [file pone.0055857.s003.doc]

**Table S2*.*** wRMSDs between two trajectories using different forces of 0.5, 1.0, 1.5 and 2.0 kcal·mol^-1^·Å^-2^. wRMSDs calculated on backbone atoms and all atoms are listed on the upper triangle and lower triangle of the table. Data in the parentheses are variance of wRMSD between two trajectories.

|  | B1 | C1 | C2 | C3 |
| --- | --- | --- | --- | --- |
| B1 | 0(0) | 1.44(0.19) | 1.45(0.23) | 1.65(0.31) |
| C1 | 1.45(0.28) | 0(0) | 1.44(0.25) | 1.62(0.33) |
| C2 | 1.48(0.25) | 1.43(0.26) | 0(0) | 1.55(0.34) |
| C3 | 1.66(0.35) | 1.61(0.34) | 1.53(0.42) | 0(0) |
