# Supplementary material for: Conformational Transition Pathway in the Activation Process of Allosteric Glucokinase
Source: PLoS One. 2013 Feb 7;8(2):e55857. doi: 10.1371/journal.pone.0055857 (PMC3567010; doi:10.1371/journal.pone.0055857)
Supplement: Text S1 — The nudged elastic band to locate the transition pathway fro the super-open to closed states. (DOC) [file pone.0055857.s004.doc]

**Nudged elastic band (NEB) method to locate the transition pathway**

The NEB method (1-3) to probe the transition pathway from the super-open state to the closed state of GK was carried out. In NEB, a string of replicas (or ‘images’) of the system are created and connected together with springs in such a way as to form a discrete representation of a path from the reactant configuration, **R**, to the product configuration, **P**. Minimization of the entire system, but with the end point structures (**R** and **P**) fixed, provides a minimum energy path. Each image between the end points is connected to the previous and next image by ‘springs’ along the path that keep each image from sliding down the energy landscape onto adjacent images. In NEB, the total force *F* on each image *i* is decoupled to perpendicular force and a parallel force by the tangent vector [Eq. (1)]. The perpendicular component of the force is obtained by subtracting out the parallel component of the force [Eq. (2)], where ∇*V*(*P_i_*) is the gradient of the energy with respect to the atomic coordinates in the system at image *i* and **τ** is the 3N dimensional tangent unit vector that describes the path. The parallel component of the force accounts for the artificial springs linking each image together [Eq. (3)], where *k_i_* is equal to the spring constant between images *P_i_* and *P_i-1_*, and *P* is the 3N dimensional position vector of image *i*.

*F_i_* = *F_i_*^^ + *F_i_*^⊥^ (1)

*F_i_*^⊥^ = - ∇*V*(*P_i_*) + ((*∇V*(*P_i_*))**⋅τ**)**τ** (2)

*F_i_*^⊥^ = [(*k_i+1_*(*P_i+1_* - *P_i_*)) – *k_i_*(*P_i_* – *P_i-1_*)) **⋅τ**]**τ** (3)

The super-open (PDB ID: 1V4T) and the closed (PDB ID: 1V4S) states of GK were referred as the starting and end points, respectively. The AMBER FF03 field was assigned to GK using the program AMBER 11 (4). A truncated octahedral box of TIP3P waters was added with a 10 Å buffer and counterions were added to maintain electroneutrality of the two systems. First, the steepest descent and conjugate gradient algorithm minimizations were introduced to remove bad contact in the two initial structures. Then, a series of replicates were created (an additional 18 yielding a total of 20 structures with 1V4T and 1V4S that we already have) that will stretch along the transition pathway and all be connected by springs. The backbone atoms of the systems were defined to apply NEB force decoupling, as well as the same part to RMS fit neighboring images to, to remove rotational and translational motion. The spring forces equal to 10 kcal mol^-1^ Å^-2^ was set in the heating stage from 0 to 300 K with a Langevin collision frequency of 1,000 ps^-1^, while in the next simulated annealing, equilibrium and slow cooling stages, the larger spring forces equal to 50 kcal mol^-1^ Å^-2^ was used. The simulated annealing method was used to optimize the transition pathway from the super-open state to the closed state. The detailed procedures have been delineated elsewhere (3,5).

**REFERENCES**

1. Henkelman G, Jónsson H (2000) Improved tangent estimate in the nudged elastic band method for finding minimum energy paths and saddle points. J Chem Phys 113: 9978-9985.

2. Henkelman G, Uberuaga BP, Jónsson H (2000) A climbing image nudged elastic band method for finding saddle points and minimum energy paths. J Chem Phys 113: 9901-9904.

3. Bergonzo C, Campbell AJ, Walker RC, Simmerling C (2009) A partial nudged elastic band implementation for use with large or explicitly solvated systems. Int J Quant Chem 109: 3781-3790.

4. Case DA, Darden TA, Cheatham TEIII, Simmerling CL, Wang J et al. (2010) AMBER 11, University of California: San Francisco.

5. AMBER tutorials: <http://ambermd.org/tutorials/advanced/tutorial5_amber11/>.
